# Regulation of Myogenesis by MechanomiR-200c/FoxO3 Axis

**DOI:** 10.3390/cells14120868

**Published:** 2025-06-09

**Authors:** Junaith S. Mohamed, Aladin M. Boriek

**Affiliations:** 1 Laboratory of Muscle and Nerve, Department of Diagnostic and Health Sciences, College of Health Professions, The University of Tennessee Health Science Center, Memphis, TN 38163, USA; 2 Center for Muscle, Metabolism and Neuropathology, Division of Rehabilitation Sciences, College of Health Professions, The University of Tennessee Health Science Center, Memphis, TN 38163, USA; 3 Integrated Biomedical Sciences Graduate Program, College of Graduate Health Sciences, The University of Tennessee Health Science Center, Memphis, TN 38163, USA; 4 Department of Medicine, and Integrative Physiology, Baylor College of Medicine, Houston, TX 77030, USA

**Keywords:** mechanical stretch, microRNAs, myoblasts, differentiation, MyoD

## Abstract

Cyclic mechanical stretch has been shown to inhibit myoblast differentiation while promoting proliferation. However, the underlying molecular mechanisms are not well understood. Here, we report that mechanical stretch inhibits the differentiation of mouse primary myoblasts by promoting the cell cycle program and by inhibiting the expression of the myogenic regulator MyoD. Stretch alters the miRNA expression profile as evidenced by miRNA microarray analysis. We identified miR-200c as one of the highly downregulated mechanosensitive miRNAs (mechanomiRs) whose expression level was increased during differentiation. This suggests that mechanomiRs-200c is a myogenic miRNA. Overexpression of mechanomiR-200c revoked the effect of stretch on myoblast differentiation, and the introduction of the mechanomiR-200c antagomir restored the stretch effect. This suggests that stretch blocks differentiation, in part, through mechanomiR-200c. The gene encoding the transcription factor FoxO3 is a known direct target of mechanomiR-200c. Interestingly, MyoD binds to the mechanomiR-200c promoter in differentiating myoblasts, whereas stretch appears to reverse such binding. Our data further demonstrate that the levels of mechanomiR-200c are robustly elevated during the early stage of the muscle repair process in young mice, but not in the injured muscle of aged mice. Overall, we identified a novel pathway, MyoD/mechanomiR-200c/FoxO3a, and the potential mechanism by which stretch inhibits myoblast differentiation.

## 1. Introduction

Mechanically active tissues such as skeletal and cardiac muscles sense their physical environment by translating physical forces and deformations into biochemical signals via the process of mechanotransduction, which modulates gene expression through diverse intracellular signaling pathways [1,2,3,4,5]. Mechanotransduction may regulate a wide range of cellular functions that are essential for organ development and cellular homeostasis, including apoptosis, proliferation, differentiation, and migration [6,7,8]. Defects in mechanotransduction contribute to the development of several human diseases that range from muscular dystrophies, cardiomyopathies, respiratory disfunction, and hearing loss to cancer progression and metastasis [1,4,5,6,7,8,9,10,11,12].

Skeletal muscle comprises nearly 40% of the total human body mass and is essential for daily physical activities. Mammalian skeletal muscle has a remarkable ability to regenerate itself over time or in response to exercise, muscle growth, muscle injury, or other myogenic stimuli [13]. Skeletal muscle regeneration is a highly orchestrated process that involves the activation, proliferation, and differentiation of quiescent muscle stem cells called satellite cells. Defects in any of the myogenic events disturb new muscle fiber development, leading to muscle impairment, which is often associated with a functional deficit, especially in sarcopenia, cachexia, and muscular dystrophy conditions where alterations in regenerative capacity play a crucial role in disease progression via mechanisms that remain unclear [14]. Studies from our labs and others show that mechanical stretch promotes myoblast proliferation, while it inhibits their differentiation [3,15,16]. However, the molecular mechanisms that regulate myogenesis in a mechanical environment have remained underexplored until now, particularly in sarcopenia.

The age-associated loss of muscle mass as well as loss of muscle function due to sarcopenia begins in humans as early as the fourth decade of life [17]. By their eighth decade, these individuals will lose 30–50% of their skeletal muscle mass and function, a loss that is worsened by muscle disuse in inactive aged people [18,19]. Although several pathophysiological issues can occur in the aging skeletal muscles, the decreased number of muscle fibers, the loss of cross-sectional area, and defective regeneration [17,20] are likely linked with decreased numbers of satellite cells and their reduced capacity for muscle repair [21]. In both human and animal models, satellite cell numbers decline in a fiber type-specific manner and their ability to self-renew is significantly reduced in aging muscles, which can lead to apoptosis or senescence [21,22,23,24]. Mechanical loading and contractile activity play critical roles in the regulation of muscle mass, and altered loading can lead to structural remodeling of muscle. Human and animal studies demonstrate that the capacity to sense and subsequently respond to mechanical stimuli is diminished in aged muscles [12]. However, a molecular mechanism that would link the loss of mechanotransduction and skeletal muscle regeneration in aging has not been uncovered.

MicroRNAs (miRNAs) are the most abundant and well-characterized small noncoding RNAs which act as genomic master switches to regulate protein expression and orchestrate multiple cellular pathways [25]. MiRNAs play a critical role in skeletal muscle disorders [26,27,28]. Using miRNA microarray, we pioneered the discovery of several mechanosensitive microRNAs (mechanomiRs) in muscle cells, including mechanomiR-26a, for which we identified a novel role in controlling the hypertrophy of airway smooth muscles [29]. MechanomiRs play roles in the pathogenicity of human diseases [30,31,32,33,34]. For example, we have recently shown dysregulation of the mechanomiR expression profile in the skeletal muscles of the mdm mouse, an animal model of muscular dystrophy [4].

The purpose of the current study is to determine the role of mechanomiRs and their mechanisms of regulation in myogenesis, which remain unexplored. We hypothesized that mechanical stretch promotes the proliferation of myoblasts while inhibiting their differentiation, in part, via a mechanomiR-mediated signaling pathway. Our data reveal that miR-200c is a mechanosensitive miRNA whose expression is regulated by MyoD. MechanomiR-200c promotes myoblast differentiation by inhibiting FoxO3 gene expression at the post-transcriptional level. We propose that the novel MyoD/mechanomiR-200c/FoxO3a axis may serve as a determinant of the myogenic program in skeletal muscle.

## 2. Materials and Methods

### 2.1. Mice

C57BL/6J mice purchased from The Jackson Laboratories (Bar Harbour, ME, USA) were bred in the animal facility of Baylor College of Medicine (BCM), and all animal experimental procedures were performed under a protocol approved by the BCM Institutional Animal Care and Use Committee. All mice were housed in a temperature-controlled room on a 12 h light/dark cycle, with a temperature of 23 °C and humidity of 40–60%. Mice were given ad libitum access to food and water. A total of nine male mice, including both young (3-month-old; n = 4) and aged (26-month-old; n = 5) animals, were euthanized; the gastrocnemius, tibialis anterior, and soleus muscles were collected and used as a source of primary satellite cell isolation.

To evaluate the muscle repair process in young and aged mice, we randomly assigned mice to two groups, 16 young (3-month-old) and 16 aged (26-month-old) mice, with 4 mice for each time point. We injected each mouse in the tibialis anterior muscle (TA) with 50 µL cardiotoxin (10 µM, diluted in 0.9% PBS). Contralateral TA muscles were injected with PBS and treated as controls. At each time point after injection (0, 3, 7, or 14 d), we euthanized a subgroup of the mice, harvested the TA muscle from each, and isolated myoblasts from satellite cells as described below.

### 2.2. Isolation of Satellite Cells and Differentiation into Myoblasts

For in vitro studies, we used primary myoblasts derived from satellite cells isolated from pooled mouse hind limb muscles (n = 4–5 mice) and cultured in growth medium to induce myoblasts, as described in [35]. These cells were used to design replicate experiments after obtaining a sufficient number of cells. Briefly, skeletal muscles were digested with 0.2% collagenase type II (285 U/mg, Sigma, St. Louis, MO, USA) in Dulbecco’s modified Eagle medium (DMEM; ThermoFisher Scientific, Grand Island, NY, USA) at 37 °C for 90 min, and the enzyme was inactivated with 20% fetal bovine serum (FBS) in F10 medium. Following two washes in phosphate-buffered saline, the tissue was triturated to mechanically dissociate individual muscle fibers, which were digested with 0.0125% Collagenase type II and 0.05% Dispase (1.81 U/mg, Sigma) in F10 medium at 37 °C for 30 min to release mononuclear cells. These cells were further dissociated by pipetting, centrifuged at 500 rpm for 30 s to remove debris, and the cell-enriched supernatant was filtered through 100 μm then 50 μm cell strainers. The filtered cells were counted using a hemocytometer and diluted to a total volume of 1 × 10^6^ cells per 100 μL of 2% FBS in Hank’s Balanced Salt Solution.

We subsequently used flow cytometry to isolate satellite cells from other mononuclear cell populations with antibodies specific for the satellite cell-specific surface markers β1-integrin, vascular cell adhesion molecule 1, and CD34 and for the hematopoietic and stromal cell markers spinocerebellar ataxia type 1, macrophage-1 antigen, lymphocyte antigen 76 (Ly76)/glycophorin A-associated antigen, CD31, and CD45. These antibodies were purchased from BD Biosciences (San Jose, CA, USA). The selection of primary and secondary antibodies tagged with specific fluorochromes was based on our Flow Cytometry and Cell Sorting Core facility protocol. Primary antibody incubations were performed on ice for 30 min. Cell viability was evaluated via the addition of propidium iodide and calcein blue AM (dilution 1:1000; Sigma-Aldrich) 10 min before flow cytometry.

All cells were sorted twice to maximize cell purity. The final sorted fractions were PI^−^, calcein^+^, Sca1^−^, CD31^−^, CD45^−^, Mac1^−^, Ter119^−^, β1-integrin^+^, VCam1^+^, and CD34^+^. Purified satellite cells were plated on 0.1% gelatin-coated dishes at low density for clonal analysis (500 cells/well in 24-well plates), incubated in growth medium (GM; DMEM containing 20% fetal calf serum, 100 units/mL penicillin, and 100 μg/mL streptomycin), and allowed to grow. Myoblast differentiation was induced when the satellite cells were 70–80% confluent by replacing GM with a differentiation medium (DM) that contained 2% horse serum in place of the 20% FBS.

### 2.3. Stretch Protocol

The stretch protocol was applied as described in [3]. Myoblasts were plated onto type I collagen-coated flexible-bottom plates (BioFlex plates, Flexcell International, McKeesport, PA, USA) and incubated at 37 °C in a CO_2_ incubator. After 24 h, the myoblasts were subjected to 1 h cyclic strain at 1 Hz (0.5 s of 17% stretch alternating with 0.5 s of relaxation) every 24 h for 5 days with a computer-controlled vacuum stretch apparatus (FX-4000T Tension Plus System, FlexCell International) using a vacuum pressure sufficient to generate 17% mechanical strain. Replicate control samples were maintained under static conditions with no applied cyclic strain. The stretch was applied to the myoblasts in GM or DM as stated.

### 2.4. miRNA Microarray Analysis

miRNA microarray analysis was performed as described in [29,36]. Briefly, total RNA was isolated from unstretched and stretched cells using TRIzol reagent according to the manufacturer’s protocol (ThermoFisher Scientific, Pittsburgh, PA, USA). An aliquot of each sample (10 µg) was fractionated by size using a YM-100 Microcon centrifugal filter (Millipore, Billerica, MA, USA) and analyzed by miRNA microarray in a core facility at Baylor College of Medicine using the proprietary μParaFlo microfluidic chip that contains 700 standard mature mouse miRNA probes (Sanger miRBase 14.0). We used a log_2_ scale to calculate the fold differences between groups of miRNAs that were differentially regulated by stretching. Bioinformatic and statistical analyses were performed as described in [29,36].

### 2.5. Reverse Transcription and Quantitative PCR (RT-qPCR)

RT-qPCR was performed to validate differentially regulated transcripts, as defined previously [29,36]. All RNAs were treated with TURBO DNase (ThermoFisher Scientific) before cDNA synthesis. One microgram of total cellular RNA was used with random primers to synthesize first-strand cDNA using the Maxima H Minus First Strand cDNA Synthesis Kit (ThermoFisher Scientific), according to the manufacturer’s protocol. The resulting cDNA was diluted 1:20 in a 5 µL volume and added to each qPCR reaction with 0.5 µm of diluted primers and the appropriate reagents from the SensiMix Real-Time PCR Kit from Meridian Bioscience (Memphis, TN, USA). The temperature cycle profile for the qPCR reactions was 95 °C for 15 min, followed by 40 cycles of 94 °C for 15 s, 60 °C for 30 s, and 72 °C for 30 s. Melting curve analysis was also included (one cycle of 95 °C for 1 min, 55 °C for 30 s, and 95 °C for 30 s) to verify the specificity of the amplified PCR products. The number of amplified transcripts (ΔΔCt) was estimated by using the comparative CT (ΔCt CT) method and normalized to an endogenous reference α-tubulin or U6 snRNA, relative to a calibrator. Since the ΔΔCt value is used to calculate the fold change, representing the level of gene expression in the experimental group relative to the calibrator (control), we marked each transcript vale in the ‘Y’ axis of the histogram as relative. All PCR products were verified on an agarose gel stained with ethidium bromide to discriminate between the correctly sized amplification products and the potential primer dimers.

### 2.6. Immunoblot Analysis

Immunoblot analysis was performed using the methods described in [35,36,37]. Briefly, proteins (40 µg) were resolved on 4–12% gradient SDS-PAGE gels (Thermo Fisher Scientific) and transferred to nitrocellulose membranes. The membranes were blocked with 5% fat-free milk for 1 h and incubated at 4 °C overnight with normal or phospho-specific primary antibodies specific for MyoD, p27 (Kip1), FoxO3, or α-tubulin as an internal control. Horseradish peroxidase (HRP)-conjugated anti-rabbit or anti-mouse IgG secondary antibodies were applied, and antibody binding was detected using chemiluminescence (Thermo Fisher Scientific). All antibodies used in this study were purchased from ThermoFisher Scientific (MyoD, cat. no. MA5-12902; p27 Kip1, cat. no. AHZ0452; FoxO3, cat. no. 720128; HRP goat anti-mouse IgG (H+L), cat. no. G-21040; and goat anti-rabbit IgG (H+L), cat. no. 31460).

### 2.7. Transfection

For the miR-200c mimetic experiment, cells were plated in 6-well plates in a medium containing 20% FBS, allowed to adhere for 24 h, transfected with 30 nM of miR-200c mimetics or a non-specific (NS) control miR (miRvana; ThermoFisher Scientific), and incubated at 37 °C for 24–48 h. For the RNAi experiment, the cells were transfected with 50 pmol NS-siRNA or FOXO3-siRNA (SantaCruz Biotechnology, Inc., Dallas, TX, USA). All transfections were performed using Lipofectamine RNAi MAX (ThermoFisher Scientific), according to the manufacturer’s instructions. Briefly, Lipofectamine (LFA) and the nucleic acid target (NS miR, miR mimetic, or siRNA) solutions were diluted individually in Opti-MEM I Reduced Serum Medium (ThermoFisher Scientific). The LFA and nucleic acid solutions were mixed gently and incubated at room temperature (RT) for 5–10 min. The lipid–nucleic acid complex mixtures were added gently to the appropriate wells and the cells were incubated as indicated for each experiment.

### 2.8. Luciferase Assays

The FoxO3 3′UTR reporter vector was generated as described in [29]. Briefly, a 400 bp mouse FoxO3 3′UTR sequence containing miR-200c binding sites was synthesized using PCR and cloned into the pmirGLO reporter plasmid (Promega, Madison, WI, USA). In parallel, we used the QuikChange II site-directed mutagenesis kit (Stratagene) to introduce site-specific mutations in the miR-200c binding sites on FoxO 3′UTR, as described in [38]. The cloned recombinant plasmid vectors were transformed into competent *E.coli* DH5a bacteria that were cultured to amplify the plasmid DNA using a standard routine laboratory procedure. Plasmid DNA was subsequently purified using the QIAprep Spin Miniprep Kit (Qiagen, Hilden, Germany). The primers used in PCR and mutagenesis are described in Table 1.

### 2.9. Chromatin Immunoprecipitation (ChIP) Assays

We performed ChIP assays to determine the binding activity of MyoD on the miR-200c promoter, as described in [5]. Briefly, cells were crosslinked using 10 μL 37% formaldehyde/mL medium at RT for 15 min, followed by the addition of glycine to a final concentration of 125 mM. Crosslinked cells were washed twice with phosphate-buffered saline (PBS), resuspended in PBS containing 1× protease inhibitor, and collected by centrifugation at 700× *g* at 4 °C for 5 min. The cell pellets were lysed in 1 mL of lysis buffer (1% SDS, 10 mM EDTA, and 50 mM Tris, pH 8.1) and sonicated on ice to shear DNA to an average length of 200 bp to 1000 bp. The sheared DNA was collected by centrifugation at 10,000× *g* at 4 °C for 10 min, divided into 100 μL aliquots, and precleared with protein G agarose as follows. To each of the 100 μL aliquots, we added 900 μL dilution buffer (0.01% SDS; 1.1% Triton X-100; 1.2 mM EDTA; 16.7 mM Tris-HCl, pH 8.1; and 167 mM NaCl) and 60 μL protein G agarose and incubated them at 4 °C for 1 h with rotation. The protein G agarose and any bound proteins were removed by centrifugation at 3000× *g* 4 °C for 1 min and the supernatant was transferred to a fresh tube. A 10 μL aliquot of each supernatant was removed as input material and stored at 4 °C, while the remainder of the supernatant was incubated with 5 μg of primary antibodies specific for MyoD, RNA polymerase II (positive control), or normal IgG (negative control) at 4 °C overnight with rotation. The antibody–DNA complexes were precipitated with the addition of 60 μL protein G agarose and incubation at 4 °C for 1 h and collected by centrifugation at 5000× *g* for 1 min. The pellets were washed in cold wash buffers (1 mL each) in the following order: low-salt buffer (1% SDS; 1% Triton X-100; 2 mM EDTA; 20 mM Tris-HCl, pH 8.1; 150 mM NaCl), then high-salt buffer. The protein–DNA complexes were resuspended in elution buffer (1% SDS, 100 mM NaHCO_3_), crosslinks were removed with 0.2 M NaCl and RNase A, and the DNA in the input and precipitated samples was analyzed by PCR with primers to detect the miR-200c promoter (Table 1). ChIP-specific primary antibodies were purchased from ThermoFisher Scientific: polyclonal rabbit anti-MyoD (cat. no. PA5-34645); mouse monoclonal anti-RNA Poly II C-terminal domain (CTD; cat. no. 49-1033); and Pacific Blue-conjugated polyclonal goat anti-mouse IgG (H+L), Cat. No. P31582). The primary antibodies were purchased from ThermoFisher Scientific: affinity purified monoclonal anti-MyoD (cat. no. MA5-12902), polyclonal RNA Polymerase II (cat. no. A303-835A), and normal rabbit monoclonal IgG (negative control).

### 2.10. Immunocytochemistry

At 60–70% confluence, cells were gently washed twice in PBS, then fixed and permeabilized with ice-cold methanol–acetone (1:1) at −20 °C for 5 min. After washing twice in PBS, the cells were blocked in 2% normal goat serum at RT for 1 h. The cells were incubated with a rabbit anti-tubulin primary antibody at RT for 2 h, washed with PBS, incubated with Alexa Fluor 547-conjugated goat anti-rabbit secondary antibodies at RT for 2 h, and washed again with PBS. The cells were mounted with DAPI (nuclear stain)-containing mounting media for analysis by fluorescent microscopy. The fusion index was calculated as the ratio of the number of nuclei in myocytes with two or more nuclei to the total number of nuclei [39].

### 2.11. Cell Proliferation Assays

Cell proliferation was measured using Click-iT chemistry according to the manufacturer’s instructions (ThermoFisher). Briefly, cells were incubated with 10 μM Clic-iT Alexa Fluor 488-conjugated EdU (5-ethynyl-2′-deoxyuridine, a thymidine analog) or Clic-iT Alexa Fluor 488-conjugated HPG (L-homopropargylglycine, a glycine analog) for the periods indicated in the text, then harvested, washed, and fixed with Click-iT fixative for 15 min. Cells were permeabilized with 1× saponin-based permeabilization and wash buffer for 30 min. After washing, the incorporation of EdU into nucleic acids or HPG into proteins was detected using the Click-iT Cell Reaction Buffer Kit according to the manufacturer’s instructions (ThermoFisher). Flow cytometry was used to estimate the fluorescence intensity of Alexa Fluor-488-bound EdU or HPG at each time point.

### 2.12. Statistical Analysis

The results are expressed as the mean values ± SEM. Comparisons among different groups were performed by one-way Analysis of Variance (ANOVA) or two-way ANOVA followed by Bonferroni post-testing. A *p*-value of less than 0.05 was considered statistically significant.

## 3. Results

### 3.1. Effect of Stretch on Myoblast Differentiation

**Figure 1:**
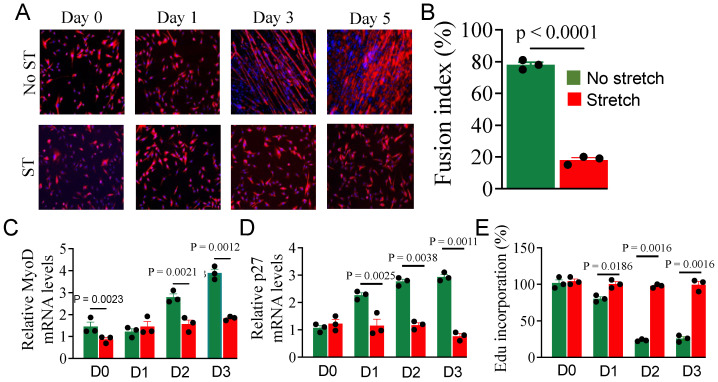
The effect of stretch on myoblast differentiation. Myoblasts cultured in GM were transferred to DM ~70–80% confluence (considered as day 1) followed by 1 h cyclic stretching for 3 or 5 days. (**A**) The cells were stained with desmin antibody and DAPI for the indicated periods and imaged at 10X under a fluorescent microscope. (**B**) The fusion index was calculated on day 5. (**C**,**D**) Total RNA was isolated from the differentiating myoblasts at the indicated periods and used in the RT-qPCR to calculate MyoD (**C**) and p27 (**D**) mRNA expression levels. Tubulin was used as a normalizer in the qPCR. (**E**) Cell proliferation was measured by % incorporation of fluorescently labeled thymidine analog (EdU) at the indicated periods. Each bar represents the mean values ± SEM (n = 3/group).

We previously showed that mechanical stretch inhibits myoblast differentiation [3], but the underlying mechanisms are poorly studied. To understand the mechanisms, myoblasts were subjected to 1-h cyclic stretching every 12 h over a 5-day period. Myoblasts in parallel cultures left unstretched were treated as controls. To assess myotube formation and differentiation, we performed immunohistochemistry using anti-tubulin antibodies and DAPI stating to calculate the fusion index. [39]. Consistent with our previous findings, stretch inhibited myoblast differentiation as evidenced by fewer myotube formations (Figure 1A) and fusion index (Figure 1B). This observation was further confirmed by decreased levels of the myogenic marker MyoD (Figure 1C) and cell cycle inhibitor protein p27 (Figure 1D) transcripts. To determine whether stretch-induced inhibition of myoblast differentiation is partially due to a failure in cell cycle exit, we measured DNA synthesis, as differentiation is typically initiated following cell cycle arrest within 24 h of induction. [40]. Stretch increased the incorporation of the thymidine analog EdU (5-ethynyl-2′-deoxyuridine) in myoblasts (Figure 1E), indicating increased DNA synthesis as a measure of increased cell proliferation. Overall, the above experimental evidence specifies that stretch inhibits myoblast differentiation but it promotes their proliferation by maintaining DNA synthesis and suppressing cell cycle inhibitors (at least p27) and myogenic regulators (at least MyoD). While our previous study showed the impact of stretch on myoblast differentiation based on cell counting [3], the present study supports such a fact by providing more evidence such as measuring cell cycle/proliferation and myogenic markers.

### 3.2. Mechanical Stretch Alters the miRNA Expression Profile

**Figure 2:**
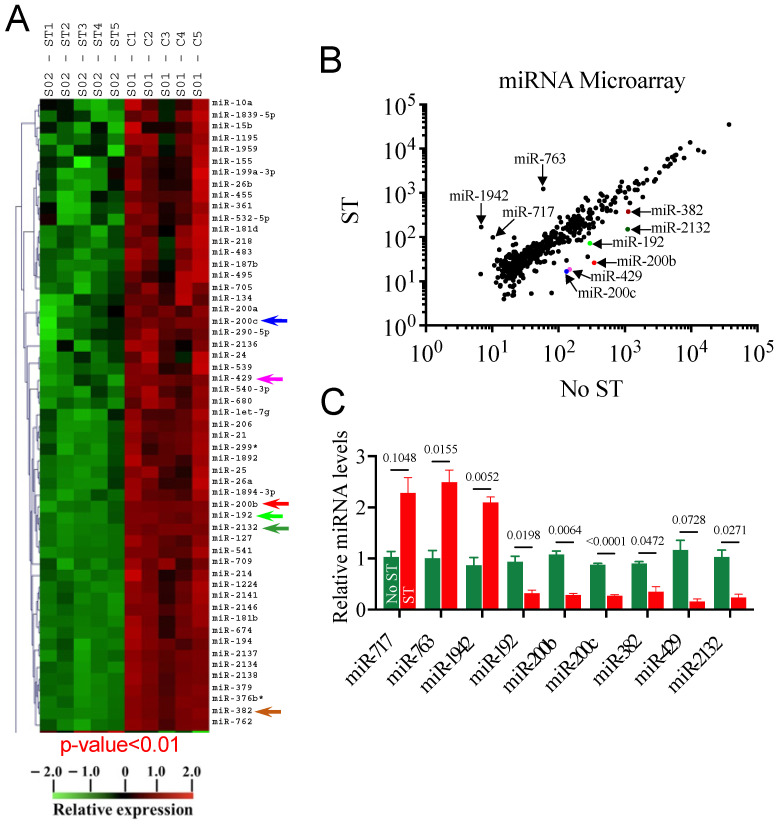
Genome-wide mechanosensitive miRNA expression profile. Myoblasts were subjected to no stretch or 1 h stretch immediately after being transferred to DM. After 12 h, total RNA was isolated and used in miRNA microarray analyses to identify differentially regulated mechanomiRs. (**A**) A heat map of miRNA microarray analysis showing differential expression of mechanomiRs in the presence (ST) or absence (**C**) of stretch. The arrows indicate the highly downregulated mechanomiRs. (**B**) A scatter plot showing log_10_-transformed signal intensities for Cy3-labeled probes in unstretched myoblasts and Cy5-labeled probes in stretched myoblasts. Each dot represents one miRNA probe. Color dots indicate the highly downregulated mechanomiRs. (**C**) A portion of the RNA used in the microarray was utilized in RT-qPCR to confirm the expression levels of the selected mechanomiRs that were differentially regulated by >1.5-fold in the microarray. Each bar represents the mean ± SEM (n = 3/group).

Studies from ours and other labs have demonstrated that stretch altered the miRNA expression profile in adult skeletal muscles and myocytes [4,15,41]. While many lines of research support the role of miRNAs in myoblast differentiation [28,42], their roles in myogenesis in a mechanical environment are poorly studied. To this end, we performed miRNA microarray screening in RNA isolated from stretched and unstretched myoblasts (control). Among 700 potential miRNAs in the array, our analyses identified nine mechanomiRs whose expression was altered by stretch in a statistically significant manner, with *p* values of 0.01 or less. Among those, stretch robustly downregulated six miRNAs (miR-192, miR-200b, miR-200c, miR-382, miR-429, and miR-2132) while upregulating three miRNAs (miR-763, miR-1942, and miR-717) (Figure 2A,B). To confirm the microarray data, we used a portion of the RNA that had been subjected to the microarray analysis in RT-qPCR, which confirmed the miRNA microarray findings (Figure 2C). U6 small nuclear RNA (snRNA), which was relatively unchanged by stretch, was used as a control and normalizer in RT-qPCR analysis. Overall, our miRNA microarray study identified a set of mechanosensitive miRNAs which may play a role in the regulation of myoblast differentiation.

### 3.3. MechanomiR-200c Inhibits Myoblast Differentiation

**Figure 3:**
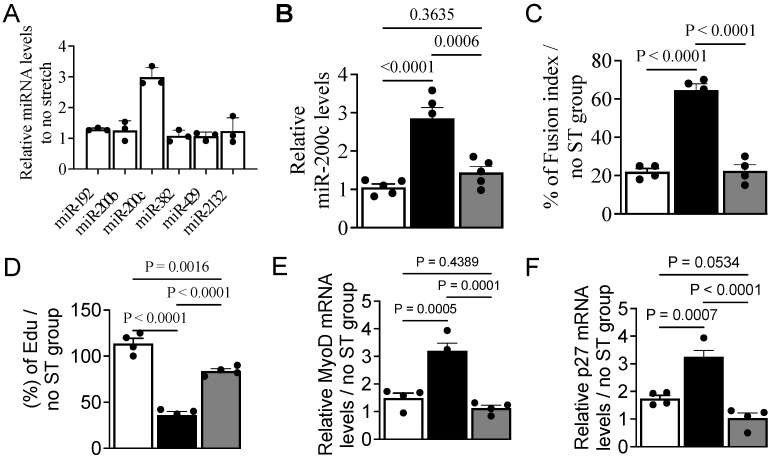
MechanomiR-200c promotes myoblast differentiation. (**A**) Total RNA was isolated from myoblasts 24 h after the switch to DM and used in RT-qPCR to determine the expression levels of mechanomiRs (n = 3). (**B**) Myoblasts growing in GM were transfected with the pEGP-miR-200c-overexpressing vector (black bar) with or without the anti-miR-200c antagomir (co-transfection; gray bar) or pEGP (empty) vector (white bar) for 24 h. Total RNA was isolated and used in RT-qPCR to determine miR-200c expression. (**C**–**F**) The transfected myoblasts were cultured in DM to induce differentiation for five days with or without stretch. Immunocytochemistry was performed on day five to calculate the fusion index (**C**). Fluorescently labeled EdU was added in the above transfected myoblasts immediately after switching to DM to determine the rate of cell proliferation on day 3 by gauging the % incorporation of EdU (**D**). Total RNA was isolated from the myoblasts and used in qPCR to determine the relative of levels MyoD (**E**) and p27 (**F**) mRNA expression. Tubulin was used as a normalizer in the qPCR. Each bar represents the fold difference of the mean ± SEM (n = 4/group) calculated according to the no-stretch group, except ‘B’.

To determine if any of the mechanomiRs identified in the miRNA microarray (Figure 2) will participate in myoblast differentiation, we isolated RNAs from myoblasts that were in DM for 12 h in the absence of stretch and were used in RT-qPCR to examine the expression levels of miR-192, miR-200b, miR-200c, miR-382, miR-429, miR-717, miR-763, miR-1942, and miR-2132. Interestingly, miR-200c was the only mechanomiR that was upregulated, with no robust change in the expression levels of the other mechanomiRs (Figure 3A). To establish the relationship between miR-200c and myoblast differentiation in a mechanical setting, we transfected myoblasts with pEGP-miR-200c overexpression or pEGP empty (control) plasmid for 24 h. Transfection of myoblasts with the pEGP-miR-200c significantly increased miR-200c levels compared to the control plasmid, and the introduction of an anti-miR-200c antagomir reduced the levels of miR-200c in the pEGP-miR-200c-transfected myoblasts near to those observed with the control (Figure 3B). This suggests that the induced expression of miR-200c is achievable, and its upregulation can be reversed in myoblasts. Myoblasts transfected with a control plasmid exhibited a fusion index of nearly 20%, whereas similar myoblasts overexpressing miR-200c displayed a fusion index of nearly 65% on day five in the presence of stretch (Figure 3C). In contrast, the introduction of the miR-200c antagomir reversed the effect of miR-200c. This suggests that miR-200c gain-of-function revokes the consequence of stretch on differentiation. Under similar stretch conditions, myoblasts transfected with a control plasmid did not show any significant changes in EdU incorporation, which contrasts with miR-200c-overexpressing myoblasts that significantly reduced EdU incorporation (Figure 3D). However, the introduction of the miR-200c antagomir reversed the miR-200c function and restored the impact of stretch on EdU incorporation. MyoD and p27 mRNA levels were robustly elevated in the presence of stretch in miR-200c-overexpressing myoblasts compared to control myoblasts. In contrast, the introduction of the miR-200c antagomir in miR-200c-overexpressing myoblasts showed the opposite effect on MyoD and p27 mRNA levels (Figure 3E,F). These results demonstrate miR-200c as a myogenic miRNA that promotes myoblast differentiation regardless of stretch stimuli, and that its downregulation is crucial for stretch-induced proliferation and inhibition of differentiation.

### 3.4. FoxO3 Is a Target Gene of miR-200c

**Figure 4:**
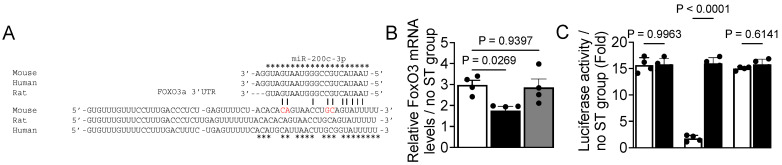
FoxO3a mRNA is a target gene of mechanomiR-200c. (**A**) Sequence alignment of the putative miR-200c targeting site in the 3′-UTR of FoxO3 shows high levels of complementarity. The star (*) indicates conserved nucleotides, while nucleotides labeled in red indicate the mutated region of the 3′UTR of FoxO3 mRNA. (**B**) Myoblasts growing in GM were transfected with the pEGP-miR-200c-overexpressing vector (black bar) with or without the anti-miR-200c antagomir (gray bar) or pEGP (white bar) vector for 24 h. Total RNA was isolated from myoblasts 48 h after switching from GM to DM with or without the stretch stimulus and was used in RT-qPCR to determine the expression levels of FoxO3a mRNA levels. (**C**) Myoblasts in GM were transfected with plasmids that contain the firefly luciferase coding region cloned downstream of a wildtype (white bar) or mutant (black bar) FoxO3a 3′-UTR in the presence of the pEGP empty vector, a pEGP-miR-200c-overexpressing vector. After 48 h of myogenic induction (switched to DM), myoblasts were collected, lysed, and used for measurement of firefly and Renilla luciferase activities in a luminometer. Firefly luciferase activities were normalized to that of Renilla luciferase and plotted. Each bar represents the fold difference of the mean ± SEM (n = 4/group) calculated according to controls (no-stretch group), except ‘C’.

To understand the mechanisms by which miR-200c regulates myoblast differentiation, we searched for predicted miR-200c target genes in the public database RNA22 [43]. Interestingly, one of the top predicted targets of miR-200c listed in the database was FoxO3, a family of transcription factors known to negatively regulate myoblast differentiation [44,45]. Moreover, the comparative sequence analysis indicates the presence of complementarity sequence homology between the miR-200c seed sequence and the 3′-UTR of FoxO3 mRNA, which is conserved among mice, rats, and humans (Figure 4A). These bioinformatic analyses provide a strong rationale to test if FoxO3 mRNA could be a target gene of miR-200c. To this end, we used myoblasts that overexpress miR-200c and the control plasmid from the experiment shown in Figure 3. Mechanical stretch on control myoblasts significantly increased FoxO3 expression, whereas miR-200c-overexpressing myoblasts reversed stretch-induced FoxO3 expression (Figure 4B). The addition of the miR-200c antagomir resulted in a statistically significant increase in FoxO3 mRNA levels. These results suggest that miR-200c targets FoxO3 mRNA.

To test whether FoxO3 mRNA is indeed a direct target of miR-200c, we used the myoblasts that overexpress miR-200c to transfect a reporter plasmid that contained the firefly luciferase gene fused to an empty vector (control), wildtype (WT) FoxO3 3′UTR cDNA, or a mutant (M) version of FoxO3 3′UTR cDNA. Myoblasts transfected with the mutated FoxO3 3′UTR cDNA or the empty vector showed luciferase activity due to the absence of complementary miR-200c binding sites on FoxO3 3′UTR (Figure 4C). In contrast, myoblasts transfected with the WT FoxO3 3′UTR reporter plasmid displayed a significant reduction in luciferase activity due to the existence of complementary miR-200c binding sites on FoxO3 3′UTR. Interestingly, the introduction of the miR-200c antagomir to myoblasts transfected WT FoxO3-3′UTR-Luc resulted in increased levels of luciferase activity that were comparable to those observed with the mutated FoxO3 3′UTR cDNA and empty vector (Figure 4C). These data suggest that FoxO3 mRNA contains a predicted miR-200c binding site in its 3′UTR and is a direct target gene of miR-200c.

**Figure 5:**
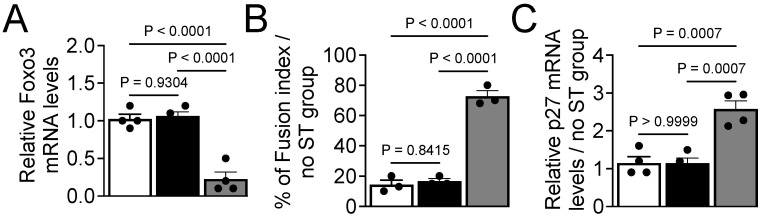
FoxO3 is necessary for the inhibitory effect of stretch on myoblast differentiation. (**A**) Myoblasts growing in GM were transfected with FoxO3 siRNA (gray bar), non-specific siRNA (black bar), or no transfection (white bar) for 24 h. Total RNA was isolated from myoblasts and used in RT-qPCR to determine the expression levels of FoxO3a mRNA levels. (**B**) The above transfected myoblasts were transferred from GM to DM to induce differentiation for five days with or without stretch. Immunocytochemistry was performed on day five to calculate the fusion index. (**C**) Total RNA was isolated from the above transfected myoblasts and used in the immunoblot assay to determine the relative levels of p27 mRNA expression on day five. Tubulin mRNA expression levels were used as a normalizer in the qPCR. Each bar represents the fold difference of the mean ± SEM (n = 4/group) calculated according to controls (no-stretch group), except ‘A’.

Next, to define the role of FoxO3 in stretch-induced inhibition of myoblast differentiation, we used a siRNA strategy to knock down the expression of FoxO3 in myoblasts and measured the induction of differentiation. We observed that siRNA-mediated inhibition of FoxO3 significantly decreased FoxO3 mRNA levels (Figure 5A), confirming the knockdown efficiency. FoxO3 knockdown induced differentiation in the presence of stretch, as evidenced by an increased fusion index (Figure 5B) and the expression of p27 mRNA (Figure 5C) compared to control and non-specific-siRNA-transfected groups. These data demonstrate clearly that the stretch-induced inhibition of myoblast differentiation occurs, in part, through the FoxO3-mediated signaling pathway. Since FoxO3 is downstream of mechanomiRs-200c, it is rational to propose that the miR-200c/FoxO3 axis is responsible for the stretch-induced inhibition of myoblast differentiation.

### 3.5. Expression of MiR-200c Is Under Transcriptional Control of MyoD

**Figure 6:**
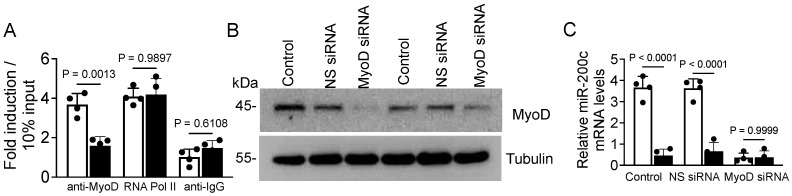
MyoD binds to the miR-200c promoter. (**A**) Myoblasts were cultured for 24 h in DM, subjected to either stretch (black bar) or no stretch (white bar), and chromatin was isolated and precipitated with anti-MyoD, anti-RNA polymerase II (positive control), or non-specific anti-IgG (negative control) antibodies. Occupancy on the miR-200c promoter by MyoD and on the GAPDH promoter by RNA polymerase II was determined using qPCR with primers specific for the miR-200c or GAPDH promoter. (**B**) Myoblasts growing in GM were transfected with MyoD siRNA, non-specific siRNA, or no transfection for 24 h. Cells were harvested and lysed, and MyoD protein levels were determined by an immunoblot assay. The gel images shown are representative of three independent experiments. (**C**) The above transfected myoblasts were cultured in DM for three days with (black bars) or without (white bars) stretch. Total cellular RNA was isolated and the levels of miR-200c were determined by RT-qPCR. Tubulin mRNA expression levels were used as a normalizer in the qPCR or loading control in the immunoblot assay. Each bar represents the fold difference of the mean ± SEM (n = 4/group) calculated according to controls (no-stretch group).

To investigate the mechanism of regulation of miR-200c, we performed a bioinformatics search with the public software PATCH (available at https://gene-regulation.com/pub/programs.html#patch; accessed on 4 April 2024) to find a transcription factor that is predicted to bind to and activate the miR-200c promoter. This analysis focused our attention on the basic helix–loop–helix transcription factor MyoD which is critical for myogenesis and whose expression in myoblasts can be suppressed by stretch [46,47,48,49]. We identified many potential MyoD binding sites on the miR-200c promoter. To determine whether MyoD can bind on the miR-200c promoter, we performed ChIP assays using antibodies specific for MyoD, RNA polymerase II (positive control), and non-specific IgG (negative control). In differentiating myoblasts on day 3 in the absence of stretch, we detected that MyoD bound to the miR-200c promoter, while stretching led to a statistically significant reduction in the level of MyoD occupancy on the miR-200c promoter (Figure 6A). As expected, there was no change in the levels of RNA polymerase II bound to the GAPDH promoter (positive control) in myogenic myoblasts regardless of stretch, suggesting the authentication of the ChIP assay. These data suggest that MyoD can bind to the miR-200c promoter possibly for transcription in the absence of stretch. To define the functional significance of MyoD interacting with the miR-200c promoter, we knocked down MyoD in myoblasts using the siRNA strategy (Figure 6B). Interestingly, myoblasts with reduced levels of MyoD were unable to express miR-200c relative to non-specific-siRNA-transfected or non-transfected myoblasts that express high levels of MyoD (Figure 6C). These data suggest that MyoD regulates miR-200c expression and such regulation is suppressed by stretch during myoblast differentiation.

### 3.6. Aging Impairs Expression of MiR-200c During Muscle Repair in Mice

**Figure 7:**
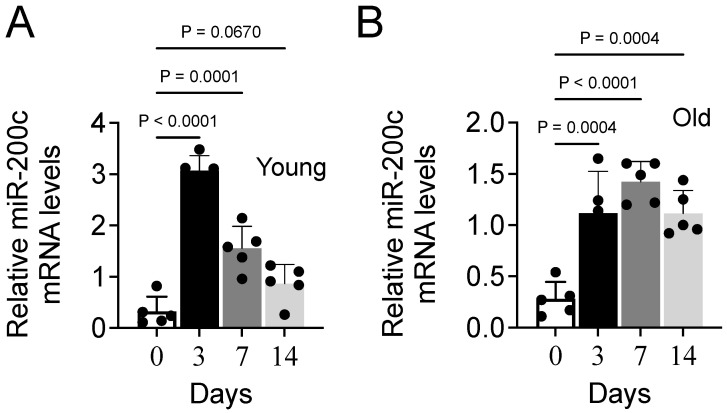
The expression of miR-200c during the muscle repair process in young and aged mice. (**A**,**B**) The tibialis anterior muscle of young and aged mice was injected with cardiotoxin to induce injury. At various time points after injury (0, 3, 7, or 14 days), the mice were euthanized, and their tibialis anterior muscles were collected. Total cellular RNA was isolated from each muscle and the levels of miR-200c in young (**A**) or aged (**B**) mice were determined using RT-qPCR. Each bar indicates the mean values ± SEM (n = 4 for young and 5 for aged mice).

To investigate the regulation of miR-200c during the muscle repair process, we used an established muscle injury model. We injected cardiotoxin into the TA muscle of young and aged mice to induce muscle damage and the subsequent repair process followed by the isolation of muscles at various periods (0, 3, 7, or 14 d). miR-200c transcripts were measured at each time point. We observed that in young mice, miR-200c levels were significantly increased 3 d after injury but decreased rapidly on days 7 and 14 after injury (Figure 7A). In aged mice, the basal expression level of miR-200c increased significantly on day 3, as observed in the young mice, albeit at a lower level, and remained higher than basal levels on days 7 and 15 (Figure 7B). Thus, in aged mice, miR-200c expression was still induced during the repair of muscular injury but to a lesser extent than that observed in young mice. This suggests that the reduction in miR-200c expression in aged muscle may result from age-related delays or dysfunction in the muscle repair process.

## 4. Discussion

In this study, we tested the hypothesis that mechanical stretch promotes myoblast proliferation and inhibits their differentiation via a mechanomiR-mediated pathway.

To test this hypothesis, we performed a comprehensive microarray analysis of miRNA expression on mechanically stretched and unstretched primary myoblasts derived from C57BL/6J mouse hind limb muscle satellite cells. We found that mechanical stretch significantly altered the miRNA expression profile in myoblasts, relative to unstretched myoblasts, and identified several mechanomiRs whose expression levels were highly modulated in response to stretch. We also found that stretch inhibited myoblast differentiation and that the inhibitory effects of stretch were mediated, in part, through mechanomiR-200c. Using extensive bioinformatics analyses followed by gain- and loss-of-function experiments, we identified the FoxO3 gene as a target of mechanomiR-200c. Furthermore, we determined that the myogenic transcription factor MyoD can bind to the mechanomiR-200c promoter. Interestingly, we detected dysregulation of miR-200c levels in aging muscle during the muscle repair process. Overall, our findings reveal a novel role for mechanomiR-200c in myoblast differentiation and suggest that its dysregulation may contribute to an impaired postnatal muscle regeneration process, particularly in response to aging.

Accumulating evidence shows a role for miRNAs in the regulation of myogenesis, especially in myoblast differentiation [28]. Our earlier study revealed that mechanical stretch promotes the proliferation of myoblasts while inhibiting their differentiation [3]. However, the underlying molecular mechanisms remained poorly studied until now. Our earlier studies demonstrated that miRNAs could respond to stretch in skeletal muscle and myocytes [3,4], suggesting that stretch-responsive miRNAs (mechanomiRs) may play a key role in the regulation of stretch-associated skeletal muscle growth and development. In the present study, we identified mechanomiR-200c as a myogenic miRNA whose expression was highly suppressed following stretch that inhibited the differentiation process. Interestingly, overexpression of mechanomiR-200c reversed the effect of stretch on differentiation, suggesting the existence of a regulatory loop between mechanomiR-200c and stretch in myoblast differentiation.

To determine whether this regulatory role of mechanomiR-200c is stretch-dependent or independent, we measured its expression during myoblast differentiation in the absence of mechanical stretch. Interestingly, mechanomiR-200c expression increased during differentiation, while this process was revoked by blocking the mechanomiR-200c function with the miR-200c antagomir. These data suggest that mechanomiR-200c positively regulates myoblast differentiation independently of stretch. Although earlier studies, including those published by us, showed a negative role for stretch in myoblast differentiation [2,3,15,41], the current study highlights a possible mechanism by which this might occur and has identified mechanomiR-200c to play a role.

FoxO3 transcription factors belong to the large forkhead family of transcriptional regulator proteins characterized by a conserved DNA binding domain, the forkhead box [50]. The FoxO3 subgroup is composed of the FoxO1, FoxO3, FoxO4, and FoxO6 proteins. FoxO1, FoxO3, and FoxO4 are expressed in most mammalian tissues [51,52]. FoxO1 and FoxO3 regulate the expression of catabolic genes in mammalian skeletal muscle [53,54,55]. The activity of the FoxO proteins is mainly regulated by phosphorylation catalyzed primarily by kinases in the PI3K–AKT signaling pathway. Phosphorylated FoxO proteins in the cytoplasm are inactive, while dephosphorylated FoxO proteins translocate to the nucleus where they are transcriptionally active [56]. FoxO3 has been shown to promote cell proliferation and inhibit differentiation in chicken myoblasts [57]. Other studies revealed negative roles for FoxO transcription factors, including FoxO3, in the regulation of myoblast differentiation [58,59,60,61,62,63,64]. Previous work from our lab identified FoxO as a stretch-responsive gene in skeletal muscle [65]. The current study demonstrates that stretch increases levels of the FoxO3 protein and has identified the FoxO3 mRNA as a mechanomiR-200c target. We propose that the stretch-induced upregulation of FoxO3 may be due to the downregulation of mechanomiR-200c expression. This is supported by our observation of a mechanomiR-200c binding site in the 3′UTR of FoxO3 mRNA, the loss of FoxO3 mRNA expression upon miR-200c overexpression, and reporter assay experiments. Overall, the predicted interaction between FoxO3 and mechanomiR-200c highlights a potential mechanism by which myoblast differentiation is regulated in response to stretch.

Skeletal muscle development is orchestrated by four conserved basic helix–loop–helix (bHLH) transcription factors jointly known as myogenic regulatory factors (MRFs), namely myogenic differentiation factor 1 (MyoD), myogenin, myogenic factor 5 (MYF5), and myogenic regulatory factor 4 (MRF4). Since the roles of MyoD and Myf5/MRF4 in myogenesis overlap, the loss of function of one of these proteins is compensated by the function of another [47,66]. For example, previous studies have demonstrated that a loss of MyoD in myoblasts resulted in incomplete differentiation and aberrant expression of muscle-specific proteins [67]. In contrast, the crucial role of myogenin in terminal myoblast differentiation is impaired in its absence, suggesting that myogenin is essential for the development of functional skeletal muscle [68,69]. The postnatal muscle regeneration/repair process relies on quiescent satellite cells in which MyoD expression is extremely low or absent. Activation of satellite cells elevates MyoD expression levels and leads to their differentiation into myoblasts [70]. Once the process of myoblast differentiation begins, MyoD increases the transcription of myogenin and the cell cycle inhibitor p21, which in subsequently enhances the transcription of muscle contractile proteins [22]. MyoD regulates many genes related to muscle development during myoblast differentiation and controls the transcription of a wide range of muscle-related genes via recruitment of transcription factors and histone acetyltransferases [71,72]. In addition, MyoD directly regulates the transcription of myogenic miRNAs [73,74,75,76,77,78,79,80,81,82].

The present study identifies mechanomiR-200c as a new miRNA target for MyoD. We showed a binding of MyoD to the E-box region of the mechanomiR-200c promoter, thereby activating its transcription during differentiation. MechanomiR-200c transcription led to the inhibition of FoxO3 activity, causing proliferating myoblasts to exit the cell cycle and enter the differentiation program. These results raise the intriguing possibility that MyoD may promote cell cycle exit through an miRNA-mediated pathway. Although previous studies established MyoD as a stretch response gene, its role in stretch-induced myogenesis was poorly understood until now [83,84,85,86]. We demonstrated a potential new mechanism by which stretch-induced inhibition of myoblast differentiation can be mediated through the MyoD-regulated myogenic mechanomiR-200c.

**Figure 8:**
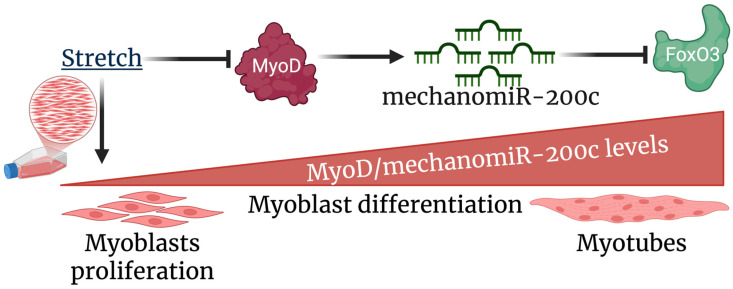
The proposed model of myogenesis following stretch.

We therefore proposed a novel model of stretch-induced inhibition of myoblast differentiation (Figure 8). In this model, induction of myogenesis in myoblasts by serum starvation results in upregulated expression of the transcription factor MyoD, which binds the mechanomiR-200c promoter to upregulate mechanomiR-200c transcription. In turn, increased levels of mechanomiR-200c target the FoxO3a mRNA by binding to its 3′UTR, thereby decreasing levels of the FoxO3 protein and promoting differentiation of myoblasts to myotubes. In contrast, cyclic mechanical stretch suppresses MyoD protein expression, thereby downregulating mechanomiR-200c expression and leading to increasing levels of FoxO3, which conserves myoblast proliferation while inhibiting differentiation.

It is important to examine the role of mechanomiR-200c in myogenesis in vivo to ensure that the proposed role of mechanomiR-200c is unique to in vitro settings or can be extended to in vivo contexts. Our muscle injury data revealed that mechanomiR-200c expression was robustly elevated in young mice muscles, especially on day 3 after muscle injury. Interestingly, the level of mechanomiR-200c expression increased at least 1.5-fold on day 7. In contrast, in aged mice, the level of mechanomiR-200c expression was below 1.5-fold between 3 and 14 days. These findings suggest that mechanomiR-200c is dysregulated in aged muscles relative to that in young mice. This could be due to the following facts. In aged mice, MyoD expression in satellite cells is impaired following muscle injury, hindering regeneration. Specifically, aging disrupts the timing and strength of MyoD binding to chromatin, leading to weaker MyoD interactions and impaired muscle regeneration [70,87]. Unfortunately, aging is associated with a loss of mechanosensitivity that can diminish muscle growth due to the low efficiency of satellite cells involved in the muscle repair process or growth [88]. These studies support our current findings that provide evidence explaining why mechanomiR-200c was downregulated in aged muscle compared to young muscle.

## 5. Conclusions

This study identifies the underlying mechanisms that regulate myoblast differentiation in response to cyclic mechanical stretch. More precisely, stretch stimulates myoblast proliferation and inhibits their differentiation by promoting cell cycle progression. The stretch-induced inhibition of myoblast differentiation is mediated, in part, through downregulation of mechanomiRs-200c, which targets the FoxO3 transcription factor, a negative regulator of myoblast differentiation. Stretch inhibits mechanomiRs-200c transcription by suppressing the MyoD protein, which binds to the promoter of mechanomiRs-200c.

## Figures and Tables

**Table 1:** Primer information.

No	Primers	Forward Sequence (5′-3′)	Reverse Sequence (5′-3′)	Purpose
1	Foxo3	CGGGCAGCCGAGGAAATGTT	TGTTGCTGTCGCCCTTATCCTT	qPCR
2	MyoD	CCTCTTTCGGTCCCTCTTTC	ATGGGTAGAGCGGCTGTAGA	qPCR
3	p27	AAGGGCCAACAGAACAGAAG	GGATGTCCATTCAATGGAGTC	qPCR
4	Tubulin	ATATCGGTCCATGTGGGTCAA	TGAGTGCCAAAGGTTCCATCC	qPCR
5	Foxo3	TGCTTGTGGTTTAGGTTCCC	ATCGGGGATGAGTAGGATAA	Mutagenesis
6	miR-200c	CTTCCGGTGCCCTTTCTCC	GGCGTCCAGCTAAGTCCTTCA	Promoter

## Data Availability

The data presented in this study are available upon request from the corresponding author.
